# Surgical management and outcomes of spinal metastasis of malignant adrenal tumor: A retrospective study of six cases and literature review

**DOI:** 10.3389/fonc.2023.1110045

**Published:** 2023-01-26

**Authors:** Xiangzhi Ni, Jing Wang, Jiashi Cao, Kun Zhang, Shuming Hou, Xing Huang, Yuanjin Song, Xin Gao, Jianru Xiao, Tielong Liu

**Affiliations:** ^1^ Department of Orthopaedic Oncology, Changzheng Hospital of the Navy Medical University, Shanghai, China; ^2^ Department of Orthopedics, No. 455 Hospital of Chinese People’s Liberation Army, The Navy Medical University, Shanghai, China; ^3^ Department of Orthopaedics, The 80th Group Army Hospital, Weifang, Shandong, China

**Keywords:** adrenal tumor, spinal metastasis, adrenocortical carcinoma, malignant phaeochromocytoma, surgery, adjuvant therapy

## Abstract

**Purpose:**

Spinal metastasis of malignant adrenal tumor (SMMAT) is an extremely rare and poorly understood malignant tumor originating from the adrenal gland. The objective of this study is to elucidate the clinical characteristics and discuss surgical management and outcomes of SMMAT.

**Methods:**

Included in this study were six SMMAT patients who received surgical treatment in our center between February 2013 and May 2022. Their clinical data and outcomes were retrospectively analyzed to gain a better understanding of SMMAT. In addition, ten cases from the literature focusing on SMMAT were also reviewed.

**Results:**

Surgery was performed successfully, and the associated symptoms were relieved significantly in all patients postoperatively. The mean follow-up duration was 26.2 (range 3-55) months. Two patients died of tumor recurrence 12 and 48 months after operation respectively. The other four patients were alive at the last follow-up.

**Conclusions:**

The prognosis of SMMAT is usually poor. Preoperative embolization and early surgical radical resection can offer satisfactory clinical outcomes. The patient’s health status, preoperative neurological function, tumor location and the resection mode are potential prognostic factors of SMMAT.

## Introduction

Adrenal tumor is a common disease seriously threatening people’s health, with the prevalence ranging from 1.0% to 8.7% with a mean of about 2% (1, 2). About 1-12% patients with adrenal tumors are diagnosed with malignancy, mainly including adrenocortical carcinoma (ACC) from the adrenal cortex and malignant phaeochromocytoma (MP) from the adrenal medulla (3, 4).

Bone metastasis is an uncommon occurrence in adrenal tumor patients but may cause poor survival prognosis. Bone metastasis accounts for about 7% in ACC patients, portending a limited survival perspective (median, 11 months) and the 5-year survival rate of MP patients is only about 40% (5–7). Especially, spinal metastasis can cause severe pain, spinal cord compression and pathological fracture, leading to poor quality of life and reduced survival (5–8).

The presence of spinal metastasis symbolizes the advanced stage of the disease in most cases. Patients in this stage are usually considered incurable and can only receive supportive care. In recent years, the important role of surgery in the treatment of spinal metastatic tumors has gradually been recognized and widely accepted (9). However, due to the rarity of spinal metastasis of malignant adrenal tumor (SMMAT) with only a few sporadic cases reported (10–15), the role and prognostic outcome of surgery for SMMAT are poorly understood. In this study, we reviewed six consecutive patients with SMMAT to present our empirical understanding about the clinical characteristics, surgical treatment, potential contributing factors for spinal metastasis, as well as prognostic factors affecting spinal overall survival of patients with this intractable malignant tumor.

## Materials and methods

This retrospective study included six patients who were pathologically confirmed as having SMMAT and received surgical treatment in our institution between February 2013 and May 2022. Hospitalization records, progress notes, surgical information, radiological presentations and pathological reports of all patients were all collected and recorded for analysis. Follow-up observation ended at the date of patient death or in May 2022. Informed consent was obtained from all participating patients before initiation of the study. The study procedures were conducted according to the principles of the Declaration of Helsinki and approved by the ethics committee of Changzheng Hospital (Shanghai, China).

All patients underwent X-ray, CT and MRI scans of the spine after admission. Positron emission tomography–computed tomography (PET–CT) was performed to detect possible metastatic sites. Tumors were further classified according to the Enneking staging system for all patients and Weinstein-BorianiBiagini (WBB) classification system for mobile spine based on radiological findings. Frankel grade and Eastern Cooperative Oncology Group performance score (ECOG-PS) were used to evaluate the neurological status and performance status, respectively. All patients had a history of adrenal tumor, and the diagnosis of metastatic adrenal tumor was confirmed by imageology. Although we emphasized the significance of percutaneous needle biopsy, some patients with serious neurological symptoms refused to have a needle biopsy for fear of aggravation of the existing condition.

Surgery was performed in all six patients successfully. Individualized adjuvant therapy was designed and implemented in each patient. X-ray and/or MRI examination was performed during the follow-up period. The last status of the patients was obtained through telephone interviews.

Studies related to SMMAT were searched by using PubMed as the searching engine, and 10 case reports were selected. Data from our own patients and those from literature research were compared and analyzed.

## Results

### Epidemiology and clinical presentation

The clinical data of the six patients in our series are listed in **Table 1**. They included of four men and two women, ranging in age from 13 to 71 years at diagnosis, with a mean of 42.2 years and a median of 41 years. The most common symptoms were radiating pain and movement disorder. Of them, two patients presented with paraplegia, and the remaining four patients had varying degrees of pain and decreased muscle strength. Four patients with pheochromocytoma had uncontrolled hypertension. The mean time from adrenal tumor resection to diagnosis of spinal metastasis was 77.5 (rang 9-216) months. The mean duration of the symptoms was 3.3 (range 1-12) months. The adrenal tumors (two cases of ACC and four cases of MP) in these patients were mainly located in the lumbar vertebrae in three cases, the thoracic vertebrae in two cases, and the sacral vertebra in one case. The Frankel scores are as follows: Grade B, Grade C and D each were documented in two patients.

**Table 1:** Clinical data of patients with SMMAT.

NO	Age/Sex	DOS (months)	Pathology	Location	Time of spinal metastasis	Tumor Size (cm)	WBB	F-S pre	ECOG pre	Operation	F-S post	Complication	Follow-up (months)	Adjunctive therapy	LR/meta	Last status
1	29/F	1	ACC	L1	120	16.8	10-12 A-E	D	1	TPR	D	None	3	Radiotherapy	None	Alive
2	71/F	1	ACC	L1-L2	9	3.7	5-10 A-D	D	2	SR	D	None	12	Radiotherapy	SM	Dead
3	13/M	1	MP	T3	24	4.6	1-4 A-D	B	3	TPR	E	None	32	None	SM	Alive
4	58/M	3	MP	S1-S3	24	1.0	1-12 A-D	C	1	TPR	E	Infection	55	None	None	Alive
5	40/M	12	MP	T3	216	5.2	5-12 A-D	C	3	TPR	D	None	48	Radiotherapy	SM	Dead
6	42/M	2	MP	L1-L2	72	0.7	1-7 A-D	B	3	TER	E	None	7	Radiotherapy	None	Alive

SMMAT, spinal metastasis of malignant adrenal tumor; DOS, duration of symptom; WBB, Weinstein-BorianiBiagini; F-S pre, preoperative Frankel scores; ECOG pre, preoperative Eastern Cooperative Oncology Group; F-S post, postoperative Frankel scores; LR/meta, local recurrence/metastasis; M, male; F, female; ACC, adrenocortical cancer; MP, malignant pheochromocytoma; TER, Total en-bloc resection; TPR, Total piecemeal resection; SR, Subtotal resection; SM, systemic metastasis.

### Imaging assessment

All patients underwent MRI and CT scans. Imageologically, SMMAT presented low signals on T1-weighted imaging and moderate signal on T2-weighted imaging on MR imaging, and on CT imaging, the diseased vertebral body presented osteolytic changes with obvious enhancement at the edge of the lesion.

### Treatment

**Figure 1:**
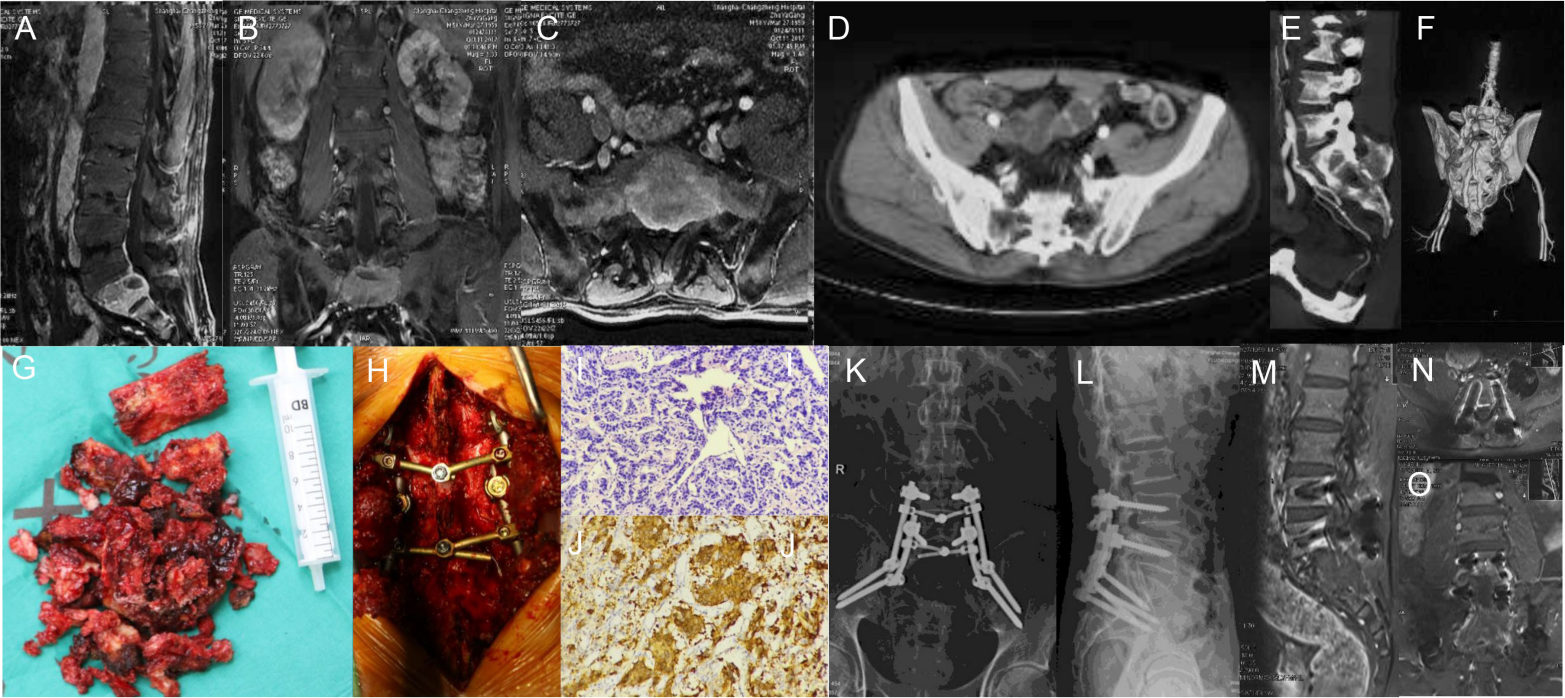
**(A–C)** Preoperative sagittal, coronal, and transverse MRI images of S1-2 vertebral body tumors; **(D–F)** Preoperative transverse and sagittal 3D CT images of S1-2 vertebral body tumors; **(G, H)** He had a history of adrenal tumor (4 points). Visceral metastases were not detectable (0 point). Bone metastases were isolated (1 point). His total prognostic score was 5 points. So, we chose the surgical strategy of total piecemeal resection. The tumor was excised by total piecemeal resection, pedicle screws, iliac screws and titanium rods were used to reconstruct the stability; **(I)** Hematoxylin-eosin (H&E) staining of pheochromocytoma; **(J)** Immunohistochemical staining for NSE; **(K, L)** Postoperative X-ray; **(M–O)** Postoperative sagittal, transverse, and coronal MRI.

The treatment process of all patients was implemented in a multi-disciplinary team (MDT) mode. The surgical indications are as follows: 1) patients with a life expectancy of more than 3 months; and 2) patients with intractable pain, spinal cord compression, and impending pathological fractures. All surgical procedures were performed by the same surgical team. The whole operation process comprised tumor excision, decompression of the spinal cord, reconstruction, and stabilization of the spine *via* a posterior approach. The surgical modality included total en-bloc resection in one patient (Case 6), subtotal resection in one patient (Case2), and total piecemeal resection in the other four patients. The vertebral defects were repaired by the titanium filled with the bone allograft in Case 3, and the titanium filled with the bone cement in Case 5. Vertebral bone cement fixation was applied in Case 1 and 2. Pedicle screws were applied in Case 4 and Case 6 (**Figure 1**). Intraoperative blood loss ranged from 500 to 4000 (mean 2050) ml. The mean duration of surgery was 312.5 (range: 200-490) min. All patients recovered well without significant surgical complications except one patient (Case 4) who developed infection, which was cured after antibiotic therapy. Pain was relieved and muscle strength was improved postoperatively in all patients. After multidisciplinary evaluation, individualized treatment plans were performed in each patient. Four patients (Case 1, 2, 5 and 6) received adjuvant radiotherapy, and the other two patients refused adjuvant radiotherapy for financial reasons.

### Follow-up observation

**Figure 2:**
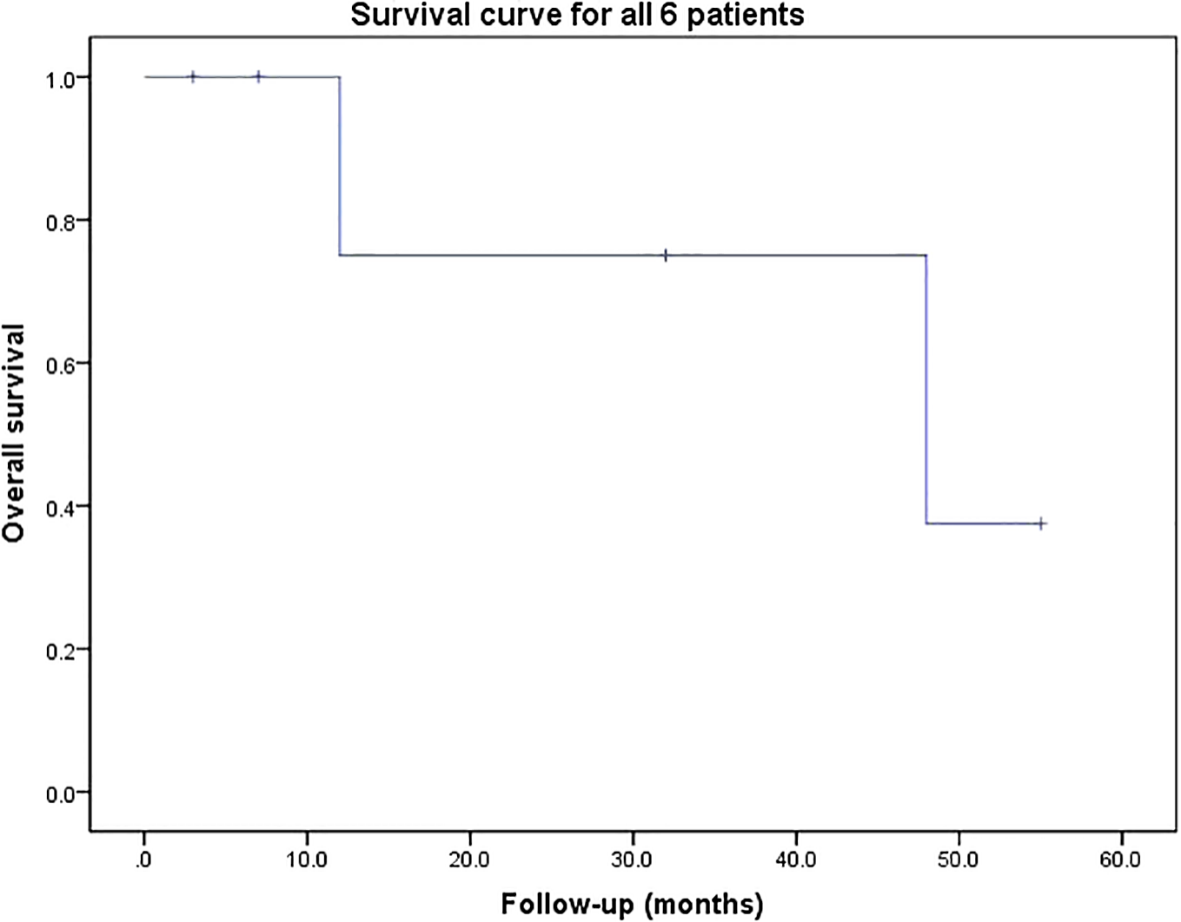
Survival curve for all 6 patients. The median survival of the six patients was 48 months.

The mean follow-up duration was 26.2 (range: 3-55) months. Pain and numbness of the lower limbs were significantly relieved in all patients after operation. Two patients (Case 3 and 6) who were unable to ambulate (Frankel grade B) preoperatively became ambulatory postoperatively. Three patients (Case 2, 3 and 5) experienced systemic tumor recurrence. Two patients (Case 2 and 5) died at the last follow-up of 12 and 48 months after operation, respectively. The others were still alive at the last follow-up. The result of survival curve for all 6 patients are shown in **Figure 2**. The median survival of the six patients was 48 months.

### Cases illustration

#### Case 1

**Figure 3:**
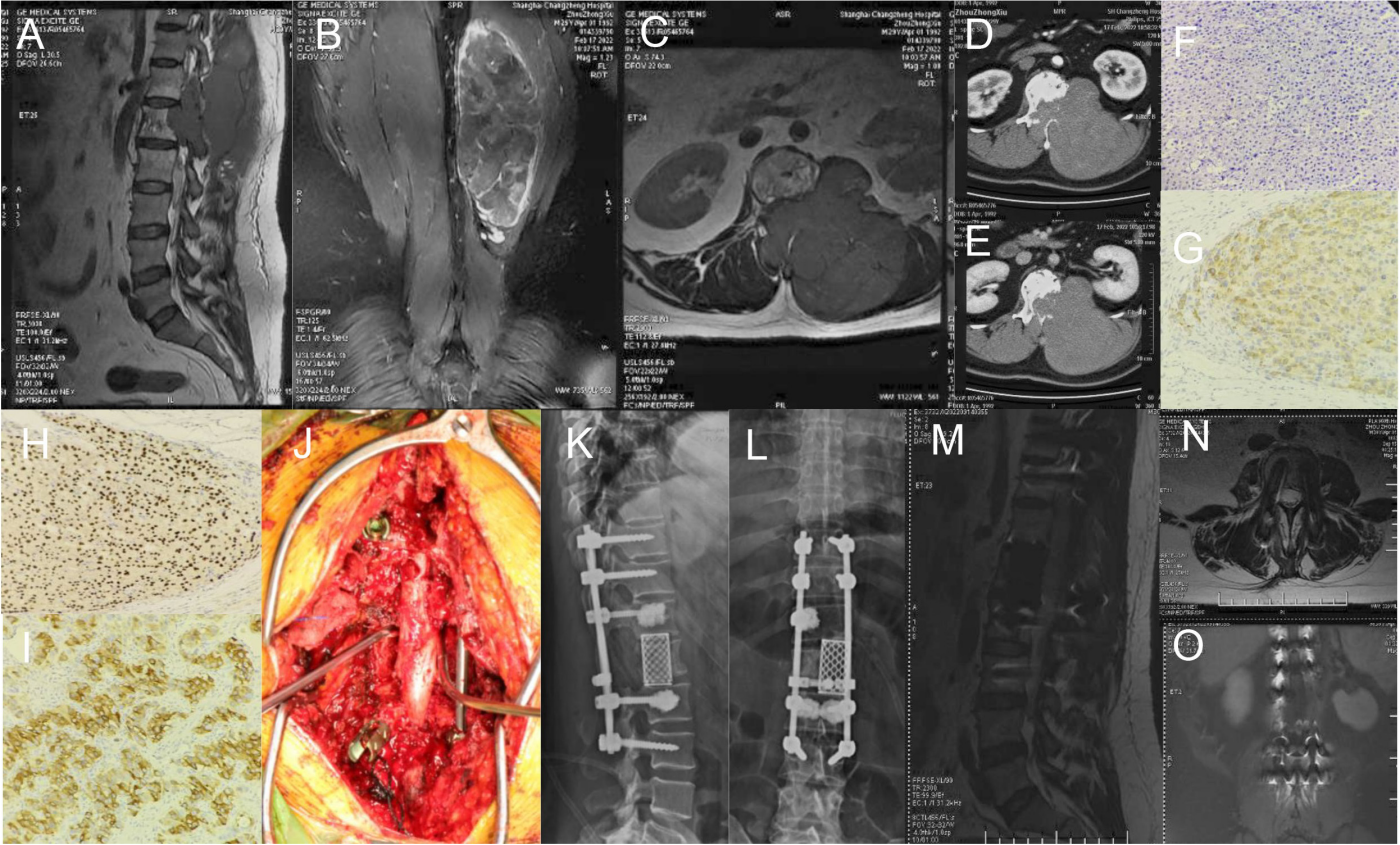
**(A–C) ** Preoperative sagittal, coronal, and transverse MRI images of L1 vertebral body tumors; **(D, E)** Preoperative transverse CT scan showed L1 vertebral body tumors; **(F)** H&E staining of adrenocortical carcinoma; **(G–I)** Immunohistochemical stainings for Inhibin a, SF-1 and Syn; **(J)** She had a history of adrenal tumor (4 points). Visceral metastases were not detectable (0 point). Bone metastases were isolated (1 point). Her total prognostic score was 5 points. So, we chose the surgical strategy of total piecemeal resection. The tumor was excised by total piecemeal resection, pedicle screws, titanium mesh and titanium rods were used to reconstruct the stability; **(K, L)** Postoperative X-ray; **(M–O)** Postoperative sagittal, transverse, and coronal MRI.

A 29-year-old woman presented with decreased muscle strength in the right leg in February 2022. Imaging demonstrated lesions in L1 (**Figure 3**). The patient’s preoperative Frankle score was D. An operation was subsequently performed to prevent further deterioration of the disease. Postoperative pathology confirmed adrenocortical carcinoma. The patient’s postoperative Frankle score was D. Patients received radiotherapy in other hospitals after surgery. The patient underwent adrenal tumor resection at local hospital in 2012.

#### Case 2

A 71-year-old woman presented with low-back pain in November 2018. Imaging demonstrated lesions in L1-L2 (**Supplementary Figure 1**). The patient’s preoperative Frankle score was D. Postoperative pathology confirmed adrenocortical carcinoma. The patient’s postoperative Frankle score was D. Patients received radiotherapy in other hospitals after surgery. And the patient underwent adrenal tumor resection at local hospital in February 2012.

#### Case 3

A 13-year-old boy received pheochromocytoma resection in 2017. The patient came to our hospital for lower limb mobility disorder in September 2019. Imaging demonstrated lesions in T3 (**Supplementary Figure 2**). The patient’s preoperative Frankle score was B. Postoperative pathology confirmed pheochromocytoma. The patient’s postoperative Frankle score was E. And he didn’t receive other adjunctive therapy.

#### Case 4

A 58-year-old man received pheochromocytoma resection in 2015. He came to hospital for sacrococcygeal pain. Imaging demonstrated lesions in S1-S2 (**Figure 1**). The patient’s preoperative Frankle score was C. Postoperative pathology confirmed pheochromocytoma. And pain was significantly relieved after operation. The patient’s postoperative Frankle score was E. He didn’t receive other adjunctive therapy.

#### Case 5

A 40-year-old man received pheochromocytoma resection in 1995. He came to our hospital for lower limb mobility disorder in February 2013. Imaging demonstrated lesions in T3. The patient’s preoperative Frankle score was C. Postoperative pathology confirmed pheochromocytoma. The patient’s postoperative Frankle score was D. He received radiotherapy in other hospitals after surgery.

#### Case 6

A 42-year-old man received pheochromocytoma resection in 2002. He was diagnosed with spinal metastasis of malignant adrenal tumor in 2008 and underwent surgical resection. He came to our hospital for lower limb mobility disorder in September 2021. Imaging demonstrated lesions in L1-L2 (**Supplementary Figure 3**). The patient’s preoperative Frankle score was B. The patient’s postoperative Frankle score was E. He received radiotherapy in other hospitals after surgery.

## Discussion

### Clinical features

Spinal metastasis of malignant adrenal tumor is a malignant tumor originating from the adrenal gland. As reported in many studies (6, 7, 16), SMMAT has a man predominance, mainly affecting individuals aged 40-60 years. ACC bone metastasis has a woman predominance with a female to male ratio of 2:1, whereas MP bone metastasis has a male predominance. The overall ratio of SMMAT is roughly the same. As ACC spinal metastasis is much rarer than MP spinal metastasis, the number of cases and reports is variable. Similar results were obtained in our center, with a man age of 42.2 years and a male to female ratio of 4:2. Most metastases of malignant adrenal tumors are more likely to occur in the thoracic vertebrae, probably due to the proximity of the adrenal gland to the thoracic vertebrae (7, 17). Due to the extremely low incidence of SMMAT, there is a lack of alertness and understanding of the disease. It is our hope that our experience, together with the patients reported in the literature, could help better understand the clinical characteristics, diagnosis and treatment of the disease.

There are no data available on the specific time from adrenal tumor resection to spinal metastasis. In our center, the mean time from adrenal tumor resection to diagnosis of spinal metastasis is 77.5 (range: 9-216) months. As the time span from adrenal tumor resection to spinal metastasis is wide and varied, it is still necessary to be alert to the occurrence of bone metastasis after adrenal tumor resection, especially spinal metastasis.

The diagnosis of patients with SMMAT represents one of the most complex and difficult problems facing clinicians. The diagnosis is usually based on pathology. In most cases of SMMAT, the clinical symptoms are caused by spinal cord compression characterized by radiating pain and varying degrees of decline in muscle strength (18, 19). Notably, two of our four MP patients with MP developed hypertension, which did not seem to be well responsive to conventional antihypertensive drug treatment. Like other spinal metastases, SMMAT is not radiographically specific (20, 21). In our case series, SMMAT presented low signals on T1-weighted imaging and moderate signals on T2-weighted MRI. On CT imaging, the diseased vertebral body showed osteolytic changes, with obvious enhancement in the edge of the lesion. All the patients in our series had a history of adrenal tumor resection. Therefore, the diagnosis of SMMAT should be considered in patients with spinal tumors who have a history of adrenal tumor resection. We recommend needle biopsy for such patients to confirm the diagnosis. Although we emphasize the importance of percutaneous needle biopsy, patients with severe neurological symptoms caused by spinal cord compression often refused to receive needle biopsy for fear of causing dissemination of cancer cells or delaying treatment.

In our center, the time from symptom onset to various degrees of muscle loss and paraplegia averaged 3.3 months for SMMAT. Compared with the 3.8-month duration of symptoms in liver cancer spinal metastasis (22), and the 4.4-month duration in clear cell renal cell carcinoma (ccRCC) (23), the progression of SMMAT seems more rapid and the degree of malignancy is relatively higher. Therefore, SMMAT is more likely to cause spinal cord compression leading to paralysis. Meanwhile, surgery for spinal metastasis is gaining acceptance, which is an effective treatment for spinal metastasis (9). In our center, all patients achieved different degrees of neurological function improvement and better mobility after surgery. Notably, thanks to rapid and timely surgical treatment, two patients (Cases 3 and 6) in our series recovered from being unable to ambulate (Frankel grade B) before operation to being able to ambulate after operation. We advocate giving patients surgery as early as possible if they are well prepared.

### Surgical strategies

In recent years, the understanding of surgery for spinal metastasis has changed. It is generally accepted that surgery could be considered when a patient has a life expectancy of more than 3 months (24). More recent evidence has shown that MDT can undoubtedly bring about better outcomes as compared with radiotherapy or chemotherapy alone, as represented by the improved quality of life after surgery (25, 26). Indications for surgery include intractable pain, spinal cord compression, and the need for stabilization of impending pathological fractures (27). The six patients in our series had definite spinal cord compression by imaging analysis and cancer pain that was difficult to be controlled by drugs. However, there are controversies over the surgical modality for spinal metastasis. In our series, total en-bloc resection was performed in one patient (Case 6), subtotal resection in one patient (Case2), and total piecemeal resection in the other four patients. Compared with patients who received subtotal resection and laminectomy (**Table 2**), patients who received total resection had a better prognosis in terms of neurological function and survival. In addition, one patient who received total en-bloc resection had better performance in local tumor recurrence control, indicating that local tumor control is important in SMMAT. For patients with a solitary lesion and surgical indications, en-bloc resection is suggested, though this tentative idea needs to be verified by more studies due to the limited number of patients in our series.

**Table 2:** Literature review for SMMAT.

No.	Author [ref.]	Age, sex	Adjunctive therapy	Time of spinal metastasis	Location	Pathology	Operation	LR/meta	Last status
1	Daniel Lee et al. (10)	55/M	Radiotherapy	5	T12	ACC	TER	None	NA
2	Drane WE et al. (11)	69/F	None	NA	L1	ACC	laminectomy	SM	Dead at 1 years
3	Ishida K et al. (12)	48/F	Chemotherapy	NA	S	ACC	None	SM	Dead at 1 years
4	Kheir E. et al. (13)	69/F	Radiotherapy	12	C6-C7	MP	SR	NA	NA
5	Yurt A et al. (14)	47/M	None	NA	T8-T9	MP	laminectomy	None	NA
6	Kaloostian PE et al. (15)	28/M	Chemotherapy	21	L3-L4	MP	SR	None	Alive
7	Kaloostian PE et al. (15)	41/M	None	36	T5-T7	MP	SR	SM	NA
8	Kaloostian PE et al. (15)	62/F	None	54	L1	MP	SR	None	Dead at 1 years
9	Kaloostian PE et al. (15)	23/M	Chemotherapy+ Radiotherapy	144	T10	MP	TER	None	NA
10	Kaloostian PE et al. (15)	21/F	Chemotherapy	NA	C7-T2,T4-T7	MP	SR + laminectomy	SM	Dead at 3 years

SMMAT, spinal metastasis of malignant adrenal tumor; LR/meta, local recurrence/metastasis; NA, not available; F, female; M, male; ACC, adrenocortical cancer; MP, malignant pheochromocytoma; TER, Total en-bloc resection; TPR, Total piecemeal resection; SR, Subtotal resection; SM, systemic metastasis.

Relevant studies present (28–30) that adrenal tumor is a hypervascular tumor with highly variable blood pressure, especially in MP. MP commonly produces one or more catecholamines, and the excess secretion of catecholamines may cause a wide array of clinical features, including hypertension (30). However, in the SMMAT patients of our series, intraoperative blood pressure did not fluctuate significantly. In addition, comparison of the surgical information of the six patients showed that the intraoperative blood loss of patients and time of surgery with preoperative embolization were lower than those of patients without embolization. Moreover, the risk factors of hemodynamic instability during the perioperative period were greatly reduced. It was reported (29, 31, 32) that preoperative embolization of spinal metastasis was safe and effective in reducing intraoperative blood loss, which also could simplify the surgical procedures. Therefore, we recommend preoperative embolization for all patients with SMMAT to ensure perioperative safety.

### Adjunctive therapy

For the adjuvant treatment of SMMAT, the team consulted about the therapeutic schedule of primary adrenal tumors. The effectiveness of radiotherapy on adrenal carcinoma has been recognized. Gonias et al. (33) affirmed the efficacy of high-dose [131I] MIBG in metastatic malignant pheochromocytoma. It was also reported that (3) adjuvant radiotherapy reduced the risk of local recurrence in ACC. However, there are controversies over the efficacy of chemotherapy for adrenal spinal metastasis. Some researchers believed (34, 35) that chemotherapy had beneficial effects on local tumor control and prolongation of survival, while others (33, 36) argued that it lacked support from large clinical trials; in addition, these trials were mostly retrospective so they could not provide direct evidence. We highlight the pros and cons of adjuvant therapy and insist that patients should have the right to choose the treatment independently. Finally, four of our six patients received radiotherapy, of whom two died of recurrence and the other two were still alive at the last follow-up. We are reluctant to make a conclusion whether or not the adjuvant therapy is effective in SMMAT due to the limited number of the patients in our series. Despite this, radiotherapy and chemotherapy remain the preferred option for advanced SMMAT at present.

### Prognosis

Although different types of adrenal tumor have the same origin, their prognoses may vary substantially. ACC seems to have the poorest prognosis, with a 5-year survival rate of 30% *vs.* 40% for MP (28, 37). Survival for spinal metastasis of ACC and MP is about 11 months (6) and 24 months (7) respectively. The median OS of the six patients in our series is 26.2 months, including the two patients who died of recurrence, which is relatively long as compared with other reports, though further statistical validation is required. On the one hand, we followed the standards of preoperative preparation strictly, and on the other hand, surgeons in the surgical team have rich experience. Compared with other carcinomas metastasized to the spine reported by our center, SMMAT showed a better prognosis than kidney cancer patients (17 months) and a poorer prognosis than liver (26.3 months) and prostate (44 months) cancer patients (22, 23, 38).

Comparison of the data between the disease-free patients (Case 1, 4 and 6) and the patients who developed recurrence or died (Cases 2, 3 and 5) showed that patients with smaller tumor sizes, a better state of health, the pathological type of MP, and better preoperative neurological function had better prognoses, suggesting that tumor size, pathological type, preoperative neurological function and the state of health may prove to be potential prognostic factors of SMMAT. However, we feel reluctant to draw a conclusion with statistical significance due to the limited number of the patients and the relatively short follow-up period. Larger-sample studies with longer follow-up periods are required to verify our findings and suggestions.

### Limitation

Our study has several limitations. First, the retrospective nature is the main limitation. Second, due to the rarity of spinal metastasis of malignant adrenal tumor, the number of cases is too small. Moreover, our study lacked a control group without surgery.

### Conclusion

SMMAT is a rare tumor with poor prognosis. The disease onset mainly presents as an osteolytic destruction. Spinal lesions can result in neurological dysfunction which seriously affecting the quality of life of the patients. The diagnosis of SMMAT is usually based on the pathology. And preoperative embolization, early surgical treatment, and total resection are usually associated with satisfactory clinical outcomes. The patient’s health status, preoperative neurological function, tumor location and the resection mode are potential prognostic factors of spinal metastasis of malignant adrenal tumor. Larger-sample, multicenter and prospective studies are required to gain deeper insights into pathogenesis, diagnosis and management of the disease.

## Data availability statement

The original contributions presented in the study are included in the article/**Supplementary Material**. Further inquiries can be directed to the corresponding authors.

## Ethics statement

Written informed consent was obtained from the individual(s), and minor(s)’ legal guardian/next of kin, for the publication of any potentially identifiable images or data included in this article.

## Author contributions

TL: Conceptualization, Supervision, Methodology. JX: Supervision, Writing- Reviewing and Editing. XG: Visualization, Investigation. XN: Formal analysis, Investigation, Writing- Original draft preparation. JC: Data curation, Writing- Reviewing and Editing. JW: Data curation, Investigation. KZ: Data curation, Investigation. SH: Investigation. XH: Investigation. YS: Investigation. All authors contributed to the article and approved the submitted version.

## Supplementary material

Supplementary Figure 1
**(A–C)** Preoperative sagittal and transverse MRI images of L1-L2 vertebral body tumors; **(D, E)** Preoperative transverse CT scan showed L1-L2 vertebral body tumors; **(F, G)** She had a history of adrenal tumor (4 points). Visceral metastases were detectable (2 points). Bone metastases were multiple (2 points). Her total prognostic score was 8 points. So, we chose the surgical strategy of subtotal resection. The tumor was excised by subtotal resection, pedicle screws, bone cement and titanium rods were used to reconstruct the stability; **(H, I)** Postoperative X-ray.

Supplementary Figure 2
**(A–C)** Preoperative sagittal, coronal, and transverse MRI images of T3 vertebral body tumors; **(D)** Preoperative transverse CT scan showed T3 vertebral body tumors; **(E, F)** He had a history of adrenal tumor (4 points). Visceral metastases were not detectable (0 point). Bone metastases were isolated (1 point). His total prognostic score was 5 points. So, we chose the surgical strategy of total piecemeal resection. The tumor was excised by total piecemeal resection, pedicle screws, titanium mesh and titanium rods were used to reconstruct the stability; **(G, H)** Postoperative X-ray; **(I–K)** Postoperative sagittal, transverse, and coronal MRI.

Supplementary Figure 1
**(A–C)** Preoperative sagittal, coronal, and transverse MRI images of L1-L2 vertebral body tumors; **(D)** Preoperative transverse CT scan showed L1-L2 vertebral body tumors; **(E)** He had a history of adrenal tumor (4 points). Visceral metastases were not detectable (0 point). Bone metastases were isolated (1 point). His total prognostic score was 5 points. So, we chose the surgical strategy of total en-bloc resection. The tumor was excised by total en-bloc resection, pedicle screws, titanium mesh, bone cement and titanium rods were used to reconstruct the stability; **(F, G)** Postoperative X-ray.
