# Supplementary figures and images for: Surgical management and outcomes of spinal metastasis of malignant adrenal tumor: A retrospective study of six cases and literature review
Source: Front Oncol. 2023 Jan 26;13:1110045. doi: 10.3389/fonc.2023.1110045 (PMC9909542; doi:10.3389/fonc.2023.1110045)

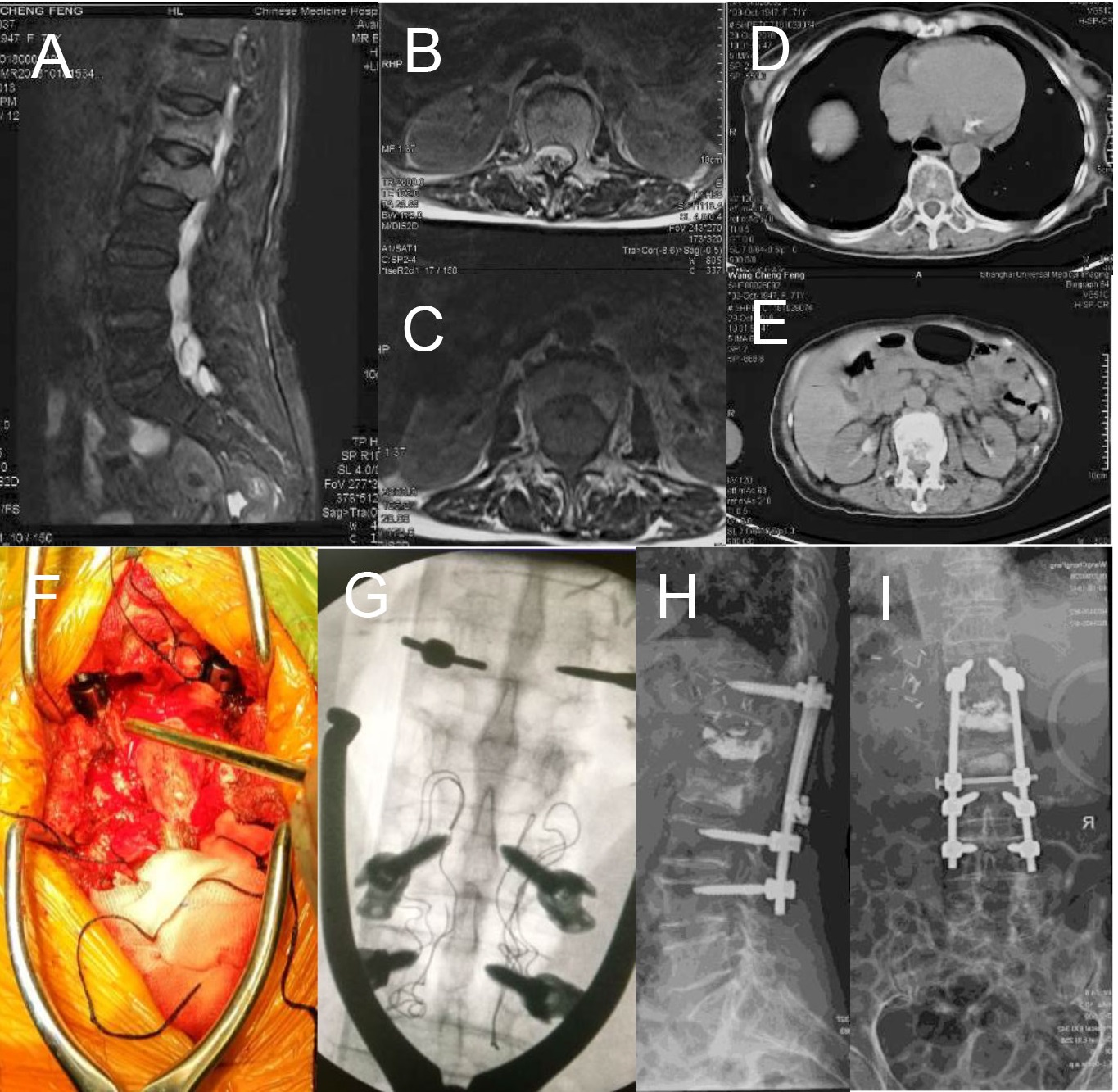

Supplement: Supplementary Figure 1 — (A–C) Preoperative sagittal and transverse MRI images of L1-L2 vertebral body tumors; (D, E) Preoperative transverse CT scan showed L1-L2 vertebral body tumors; (F, G) She had a history of adrenal tumor (4 points). Visceral metastases were detectable (2 points). Bone metastases were multiple (2 points). Her total prognostic score was 8 points. So, we chose the surgical strategy of subtotal resection. The tumor was excised by subtotal resection, pedicle screws, bone cement and titanium rods were used to reconstruct the stability; (H, I) Postoperative X-ray. [file Image_1.jpeg]

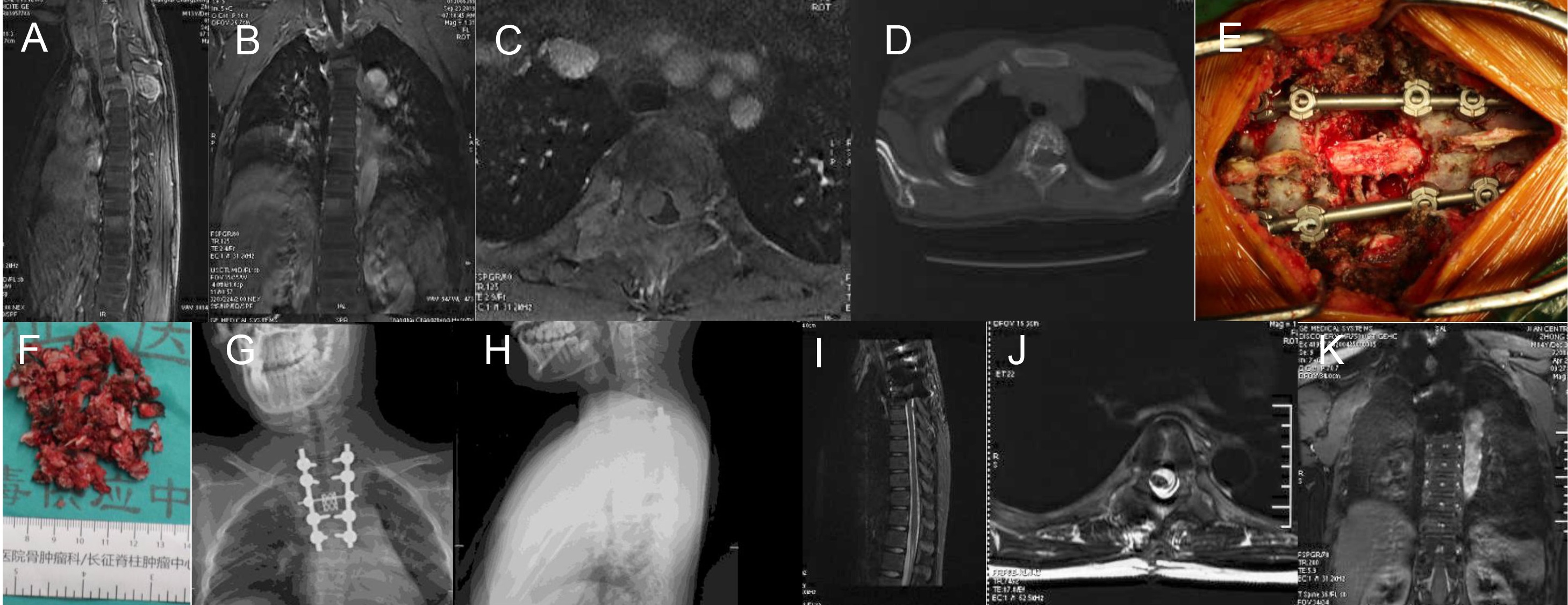

Supplement: Supplementary Figure 2 — (A–C) Preoperative sagittal, coronal, and transverse MRI images of T3 vertebral body tumors; (D) Preoperative transverse CT scan showed T3 vertebral body tumors; (E, F) He had a history of adrenal tumor (4 points). Visceral metastases were not detectable (0 point). Bone metastases were isolated (1 point). His total prognostic score was 5 points. So, we chose the surgical strategy of total piecemeal resection. The tumor was excised by total piecemeal resection, pedicle screws, titanium mesh and titanium rods were used to reconstruct the stability; (G, H) Postoperative X-ray; (I–K) Postoperative sagittal, transverse, and coronal MRI. [file Image_2.jpeg]

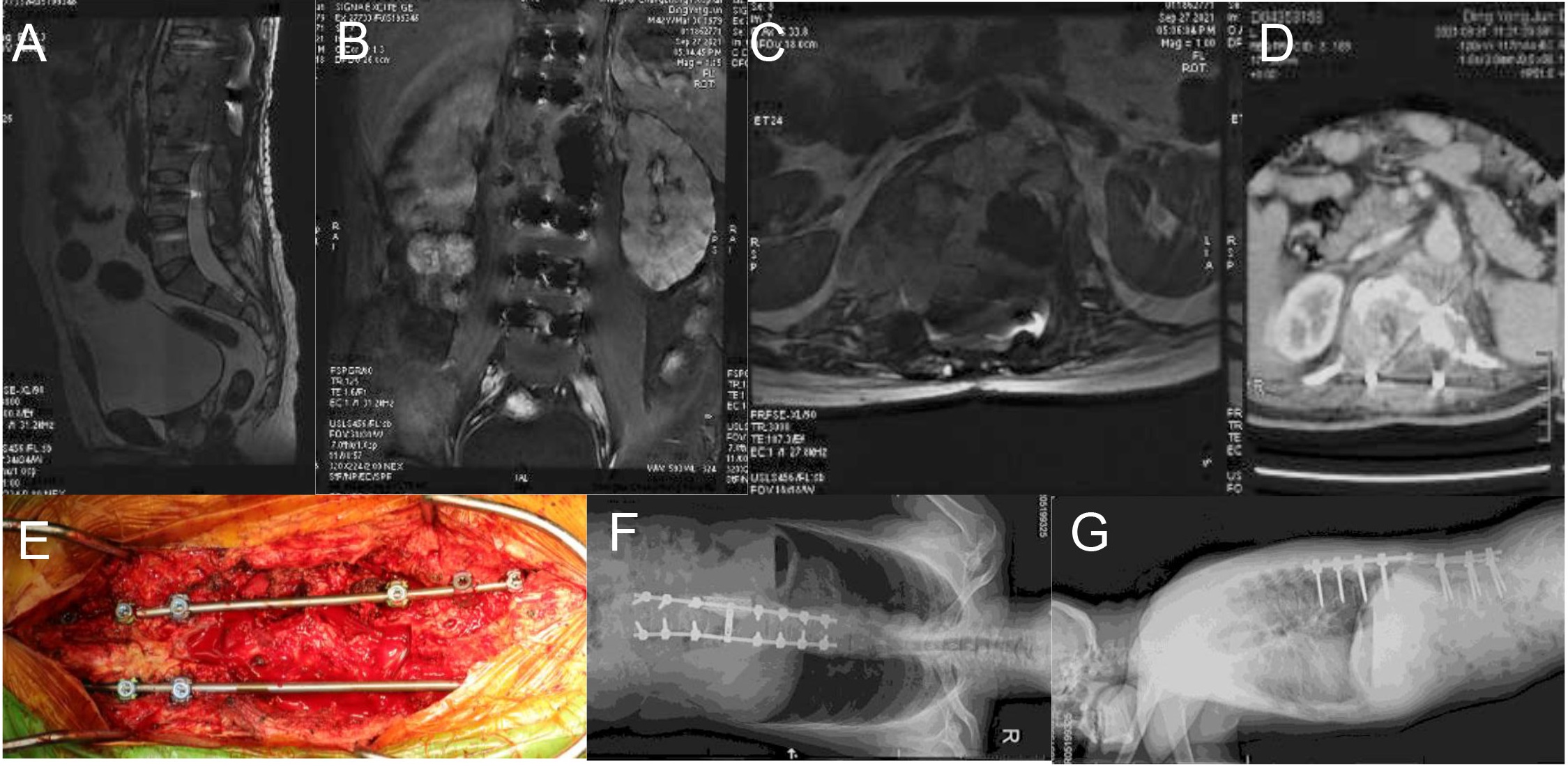

Supplement: Supplementary Figure 1 — (A–C) Preoperative sagittal, coronal, and transverse MRI images of L1-L2 vertebral body tumors; (D) Preoperative transverse CT scan showed L1-L2 vertebral body tumors; (E) He had a history of adrenal tumor (4 points). Visceral metastases were not detectable (0 point). Bone metastases were isolated (1 point). His total prognostic score was 5 points. So, we chose the surgical strategy of total en-bloc resection. The tumor was excised by total en-bloc resection, pedicle screws, titanium mesh, bone cement and titanium rods were used to reconstruct the stability; (F, G) Postoperative X-ray. [file Image_3.jpeg]
